# 8-oxoguanine DNA glycosylase (OGG1) deficiency elicits coordinated changes in lipid and mitochondrial metabolism in muscle

**DOI:** 10.1371/journal.pone.0181687

**Published:** 2017-07-20

**Authors:** Vladimir Vartanian, Jana Tumova, Pawel Dobrzyn, Agnieszka Dobrzyn, Yusaku Nakabeppu, R. Stephen Lloyd, Harini Sampath

**Affiliations:** 1 From the Oregon Institute of Occupational Health Sciences, Oregon Health & Science University, Portland, Oregon, United States of America; 2 Department of Nutritional Sciences, Rutgers University, New Brunswick, New Jersey, United States of America; 3 Nencki Institute of Experimental Biology, Warsaw, Poland; 4 Division of Neurofunctional Genomics, Department of Immunobiology and Neuroscience, Medical Institute of Bioregulation, Kyushu University, Fukuoka, Japan; 5 Department of Physiology and Pharmacology, Oregon Health & Science University, Portland, Oregon, United States of America; 6 Rutgers Center for Lipid Research and Center for Digestive Health, New Jersey Institute for Food, Nutrition, and Health, Rutgers University, New Brunswick, New Jersey, United States of America; Universidad Pablo de Olavide, SPAIN

## Abstract

Oxidative stress resulting from endogenous and exogenous sources causes damage to cellular components, including genomic and mitochondrial DNA. Oxidative DNA damage is primarily repaired via the base excision repair pathway that is initiated by DNA glycosylases. 8-oxoguanine DNA glycosylase (OGG1) recognizes and cleaves oxidized and ring-fragmented purines, including 8-oxoguanine, the most commonly formed oxidative DNA lesion. Mice lacking the OGG1 gene product are prone to multiple features of the metabolic syndrome, including high-fat diet-induced obesity, hepatic steatosis, and insulin resistance. Here, we report that OGG1-deficient mice also display skeletal muscle pathologies, including increased muscle lipid deposition and alterations in genes regulating lipid uptake and mitochondrial fission in skeletal muscle. In addition, expression of genes of the TCA cycle and of carbohydrate and lipid metabolism are also significantly altered in muscle of OGG1-deficient mice. These tissue changes are accompanied by marked reductions in markers of muscle function in OGG1-deficient animals, including decreased grip strength and treadmill endurance. Collectively, these data indicate a role for skeletal muscle OGG1 in the maintenance of optimal tissue function.

## Introduction

Endogenous metabolic byproducts and exogenous oxidants can cause extensive oxidative damage to both genomic and mitochondrial DNA (mtDNA). Unrepaired oxidative DNA damage can in turn lead to mutagenesis, cellular transformation and dysfunction, and cell death [1–4]. The cell therefore has multiple mechanisms to guard against DNA damage and to repair existing damage. The primary pathway for repair of non-bulky oxidative lesions is the base-excision repair (BER) pathway that is initiated by DNA glycosylases. These enzymes, including 8-oxoguanine DNA glycosylase (OGG1), Nei endonuclease VIII-like (NEIL)1, NEIL2, NEIL3, and endonuclease III-like 1 (NTH1), have distinct tissue distributions and substrate specificities to recognize and excise specific subsets of lesions, and in some cases, can further process the damaged site to a single-strand break via an intrinsic AP lyase activity [5–7].

8-oxo-7,8-dihydroguanine (8-oxoG) is the most commonly formed oxidative lesion in the cell. Due to its propensity to mispair with adenine during replication, it can give rise to G:C to T:A transversions and is therefore considered to be a particularly mutagenic lesion [2]. 8-oxoG is repaired by OGG1, a DNA repair glycosylase that localizes to both the nucleus and mitochondria [8–12]. OGG1 has been investigated for its role in many disease pathways, including various cancers, [5,13–19] and neurological diseases such as Parkinson’s [20–22] and Alzheimer’s disease [1,23–27]. Additionally, we previously reported that mice lacking OGG1 (*Ogg1*^*-/-*^) are prone to features of metabolic syndrome, including increased body weight and adiposity, fatty liver, elevated triglycerides, and impaired glucose tolerance [28]. Concomitantly, several groups have reported a correlation between polymorphisms in the *OGG1* gene and incidence of obesity and type II diabetes in human cohorts [29,30].

Skeletal muscle is one of the primary sites of glucose disposal in the body, and pathologies such as insulin resistance and diabetes are thought to be affected by the accumulation of lipids and alterations in the metabolic capacity of skeletal muscle. Since *Ogg1*^*-/-*^ mice were previously shown to be prone to insulin resistance, we were interested in determining the effects of OGG1 deficiency on skeletal muscle metabolism and function. In order to avoid the confounding effects of excess body weight and adiposity on muscle function, the studies presented below were carried out in chow-fed animals, prior to the onset of differences in body mass and composition.

## Results

### *Ogg1*^*-/-*^ *m*ice have increased muscle lipid deposition

We have previously shown that mice lacking the BER enzyme OGG1 are prone to obesity and insulin resistance with increasing age or upon high-fat diet (HFD) feeding [28]. Since skeletal muscle is one of the primary sites of glucose disposal in the body, we were interested in determining the effects of OGG1 deletion on skeletal muscle physiology and function. For this study, WT and *Ogg1*^*-/-*^ mice were studied under chow-fed conditions at 22 weeks of age, prior to the onset of adiposity in *Ogg1*^*-/-*^ animals.

**Fig 1:**
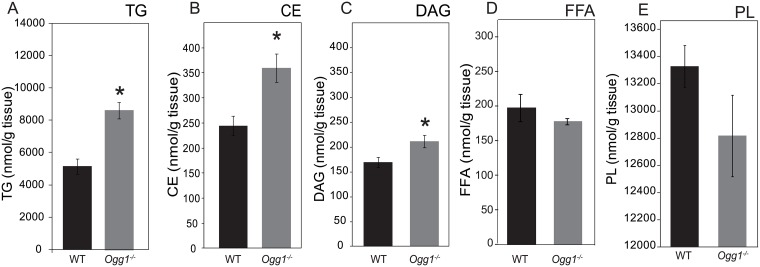
Lipid content of skeletal muscle. Total content of various lipid classes including triacylglycerol (A), cholesterol ester (B), diacylglycerol (C), free fatty acid (D), and phospholipid (E) were measured by TLC followed by gas chromatography. Data are averages of 6 animals per genotype and are presented as average nmol fatty acid per g of wet weight. Data are expressed as mean ± SEM; *p<0.05 vs. WT.

Since increased intramuscular muscle lipid deposition is a known risk factor for impaired insulin signaling [31–33], muscle lipid content was measured in WT and *Ogg1*^*-/-*^ animals. Interestingly, *Ogg1*^*-/-*^ mice had significantly increased muscle triacylglycerols (TG), cholesterol esters (CE), and diacylglycerols (DAG) (Fig 1A–1C), relative to WT counterparts. Total free fatty acid (FFA) and phospholipid (PL) content were not significantly different between the genotypes (Fig 1D and 1E). In addition to changes in total content, the fatty acid composition of several lipid moieties was also differentially affected by genotype (Tables 1–5). The increases in muscle TG and CE were primarily accounted for by an accumulation of 16 and 18 carbon mono- and polyunsaturated fatty acids, which are the predominant substrates for synthesis of these storage lipids (Tables 1–5). The observed increase in the DAG fraction in *Ogg1*^*-/-*^ muscle was primarily due to an increase in oleic acid (18:1 n-9), as well as the polyunsaturated fatty acids arachidonic acid (20:4 n-6) and docosahexanoic acid (22:6 n-3) (Table 3). Interestingly, although total free fatty acid (FFA) levels were not increased in muscle of *Ogg1*^*-/-*^ mice, levels of docosahexanoic acid (DHA, 22:6) were significantly higher in the FFA fraction of muscle lipids in these animals, relative to WT counterparts (Table 4). It is notable that this increase in free DHA in *Ogg1*^*-/-*^ mice represented an over 20-fold increase over WT levels. Most studies detailing the role of omega-3 fatty acids in skeletal muscle performance are performed under conditions of dietary supplementation with excess omega-3 lipids [34]. In our model, the *Ogg1*^*-/-*^ mice display an increase in DHA without dietary supplementation, possibly due to increased mobilization or altered partitioning of this polyunsaturated fat. The physiological significance of this dramatic increase is not yet understood. Total phospholipid (PL) content was not significantly different between WT and *Ogg1*^*-/-*^ mice. However, levels of vaccenic acid (18:1 n-7) and arachidonic acid (20:4) were significantly reduced in PL from *Ogg1*^*-/-*^ muscle, relative to WT counterparts (Table 5). This reduction in PL arachidonic acid parallels the increase in this fatty acid species within the DAG pool.

**Table 1:** Muscle triacylglycerol (TG) fatty acid composition. Fatty acid composition of triacylglycerol from gastrocnemius of WT and *Ogg1*^*-/-*^ mice was analyzed by gas chromatography. Data are averages of 6 animals per genotype and are presented as average nmol fatty acid per g of wet weight. Data are expressed as mean ± SEM; *p<0.05 vs. WT.

Fatty acid	WT (nmol/g)		*Ogg1*^*-/-*^ (nmol/g)	
	Avg	SEM	Avg	SEM
**14:0**	221.75	47.79	160.79	13.52
**16:0**	1800.67	235.55	2030.81	153.09
**16:1**	511.69	30.42	1396.56*	108.16
**18:0**	376.69	43.10	299.88	30.74
**18:1 n-9**	1145.62	55.22	2778.29*	262.82
**18:1 n-7**	168.19	22.87	261.19*	28.38
**18:2**	854.43	98.46	1584.45*	115.51
**18:3 n-6**	0.00	0.00	0.00	0.00
**18:3 n-3**	2.73	0.31	5.05	0.31
**20:0**	6.22	1.26	4.24	0.32
**20:1**	5.66	0.50	14.38*	2.04
**20:4**	37.11	6.80	44.17	6.25
**20:5**	6.21	0.64	8.39*	0.35
**22:6**	19.37	2.69	26.79	2.76
**22:0**	0.00	0.00	0.00	0.00
**24:0**	0.00	0.00	0.00	0.00
**24:1**	0.00	0.00	0.00	0.00
**Total**	5156.33	475.13	8614.99*	510.19

*p<0.05 vs WT.

**Table 2:** Muscle cholesterol ester (CE) fatty acid composition. Fatty acid composition of cholesterol ester from gastrocnemius of WT and *Ogg1*^*-/-*^ mice was analyzed by gas chromatography. Data are averages of 6 animals per genotype and are presented as average nmol fatty acid per g of wet weight. Data are expressed as mean ± SEM; *p<0.05 vs. WT.

Fatty acid	WT (nmol/g)		*Ogg1*^*-/-*^ (nmol/g)	
	Avg	SEM	Avg	SEM
**14:0**	44.90	6.60	50.84	3.29
**16:0**	45.93	4.97	78.23*	6.15
**16:1**	21.89	5.03	27.60	4.89
**18:0**	62.27	5.59	93.81*	5.95
**18:1 n-9**	27.63	2.25	50.72*	9.22
**18:1 n-7**	5.78	0.37	12.23*	2.07
**18:2**	25.08	1.15	31.29	4.03
**18:3 n-6**	0.00	0.00	0.00	0.00
**18:3 n-3**	1.12	0.27	4.04*	1.27
**20:0**	1.17	0.15	1.43	0.15
**20:1**	1.04	0.18	1.59	0.20
**20:4**	3.82	0.12	5.35	0.70
**20:5**	3.36	0.20	4.08	0.56
**22:0**	0.87	0.10	1.12	0.12
**22:6**	0.98	0.26	1.40	0.17
**24:0**	0.00	0.00	0.00	0.00
**24:1**	0.00	0.00	0.00	0.00
**Total**	244.72	18.61	359.69*	28.74

*p<0.05 vs WT.

**Table 3:** Muscle diacylglycerol (DAG) composition. Fatty acid composition of diacylglycerol from gastrocnemius of WT and *Ogg1*^*-/-*^ mice was analyzed by gas chromatography. Data are averages of 6 animals per genotype and are presented as average nmol fatty acid per g of wet weight. Data are expressed as mean ± SEM; *p<0.05 vs. WT.

Fatty acid	WT (nmol/g)		*Ogg1*^*-/-*^ (nmol/g)	
	Avg	SEM	Avg	SEM
**14:0**	2.98	0.58	3.63	0.87
**16:0**	65.51	4.43	68.75	4.03
**16:1**	5.43	0.97	4.01	1.06
**18:0**	65.02	5.05	69.03	2.99
**18:1 n-9**	2.73	0.73	24.11*	2.43
**18:1 n-7**	1.27	0.27	3.17	0.87
**18:2**	15.61	1.40	19.10	2.26
**18:3 n-6**	0.00	0.00	0.00	0.00
**18:3 n-3**	0.00	0.00	0.00	0.00
**20:0**	0.62	0.06	0.62	0.04
**20:1**	0.82	0.05	0.71	0.04
**20:4**	1.62	0.40	7.39*	1.01
**20:5**	2.66	0.16	2.54	0.15
**22:0**	0.41	0.02	0.36	0.03
**22:6**	4.75	0.48	8.31*	0.92
**24:0**	0.00	0.00	0.00	0.00
**24:1**	0.00	0.00	0.00	0.00
**Total**	169.43	9.93	211.74*	12.52

*p<0.05 vs WT.

**Table 4:** Muscle free fatty acid (FFA) composition. Fatty acid composition of free fatty acids from gastrocnemius of WT and *Ogg1*^*-/-*^ mice was analyzed by gas chromatography. Data are averages of 6 animals per genotype and are presented as average nmol fatty acid per g of wet weight. Data are expressed as mean ± SEM; *p<0.05 vs. WT.

Fatty acid	WT (nmol/g)		*Ogg1*^*-/-*^ (nmol/g)	
	Avg	SEM	Avg	SEM
**14:0**	7.37	2.28	1.92*	0.28
**16:0**	60.40	5.59	40.49*	1.29
**16:1**	4.98	1.20	4.09	1.23
**18:0**	81.28	5.64	72.63	4.75
**18:1 n-9**	18.24	4.05	17.42	2.11
**18:1 n-7**	3.78	0.45	3.80	0.09
**18:2**	15.09	2.95	15.30	1.63
**18:3 n-6**	0.00	0.00	0.00	0.00
**18:3 n-3**	0.00	0.00	0.00	0.00
**20:0**	0.81	0.07	0.64	0.04
**20:1**	0.45	0.05	0.45	0.04
**20:4**	1.13	0.12	1.03	0.04
**20:5**	2.79	0.20	2.21*	0.09
**22:6**	0.75	0.08	17.52*	1.86
**22:0**	0.46	0.06	0.45	0.04
**24:0**	0.00	0.00	0.00	0.00
**24:1**	0.00	0.00	0.00	0.00
**Total**	197.53	19.94	177.94	4.06

*p<0.05 vs WT.

**Table 5:** Muscle phospholipid (PL) fatty acid composition. Fatty acid composition of phospholipids from gastrocnemius of WT and *Ogg1*^*-/-*^ mice was analyzed by gas chromatography. Data are averages of 6 animals per genotype and are presented as average nmol fatty acid per g of wet weight. Data are expressed as mean ± SEM; *p<0.05 vs. WT.

Fatty acid	WT (nmol/g)		*Ogg1*^*-/-*^ (nmol/g)	
	Avg	SEM	Avg	SEM
**14:0**	99.50	6.37	99.76	3.84
**16:0**	6046.63	72.04	6059.67	124.83
**16:1**	228.51	8.20	254.87	15.24
**18:0**	1403.95	70.63	1229.76	54.56
**18:1 n-9**	503.86	51.13	445.45	53.56
**18:1 n-7**	450.22	11.50	402.37*	10.98
**18:2**	956.54	59.51	861.60	27.55
**18:3 n-6**	0.00	0.00	0.00	0.00
**18:3 n-3**	0.00	0.00	0.00	0.00
**20:0**	9.27	1.25	9.20	1.21
**20:1**	20.06	0.78	18.99	0.91
**20:4**	739.45	27.79	623.31*	20.72
**20:5**	53.14	3.05	48.54	2.00
**22:0**	11.28	2.27	9.22	1.54
**22:6**	2787.21	81.85	2736.65	101.56
**24:0**	8.32	1.64	9.59	1.44
**24:1**	10.22	1.78	8.59	0.69
**Total**	13328.16	153.12	12817.56	298.56

*p<0.05 vs WT.

### No change in mtDNA content or mitochondrial OXPHOS protein content in *Ogg1*^*-/-*^ mice

**Fig 2:**
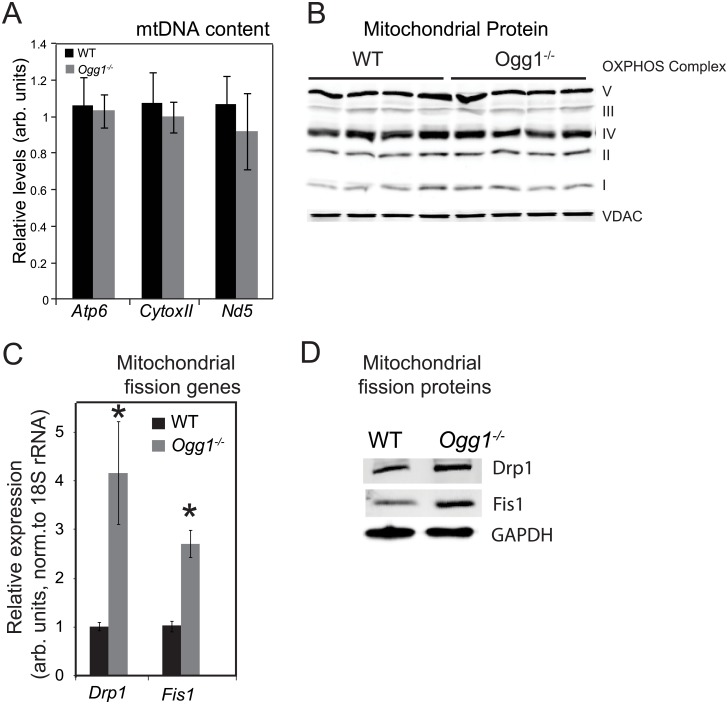
Mitochondrial DNA, protein, and gene expression. Content of mtDNA (A) was measured by qPCR using targeted primers against regions encoding for ATP synthase F_o_ subunit 6 (Atp6), Cytochrome c oxidase subunit 2 (CytoxII), and NADH dehydrogenase 5 (Nd5). Data are expressed as mean ± SEM for 6 animals in each genotype and are normalized for the high-copy number nuclear gene, 18S rRNA. Mitochondrial protein content (B) was measured by probing for proteins of oxidative phosphorylation (OXPHOS) and the voltage dependent anion channel (VDAC). Genes (C) and proteins (D) regulating mitochondrial fission, including DRP1 and FIS1 were measured by qPCR using gene-specific primers and by Western blotting, respectively. For protein gels, cropped images are presented for clarity. Full length blots are presented in S1 Fig. Data are representative of 4–6 animals per genotype and expressed as mean ± SEM; *p<0.05 vs. WT.

There is considerable disagreement in the field regarding the role of skeletal muscle mitochondrial dysfunction in the development of whole body insulin resistance [35–48]. Since we have previously reported that *Ogg1*^*-/-*^ mice have impaired insulin sensitivity [28], and given that OGG1 is involved in repair of both genomic, as well as mtDNA [5,49], we were interested in determining the role of OGG1 deficiency on mitochondrial content in the muscle of these animals. There were no differences in total content of mtDNA between genotypes, as measured by qPCR against several different mtDNA target regions (Fig 2A). Additionally, expression of proteins involved in mitochondrial OXPHOS, as well as VDAC, a mitochondrial membrane protein that is indicative of mitochondrial abundance, were not different between genotypes (Fig 2B). Collectively, these data indicate that OGG1 deficiency does not result in altered mitochondrial content in skeletal muscle. Therefore, a reduction in mitochondrial content is not likely to be a contributor to insulin resistance in the *Ogg1*^*-/-*^ model.

### No alterations in DNA damage markers in skeletal muscle of *Ogg1*^*-/-*^ mice

**Fig 3:**
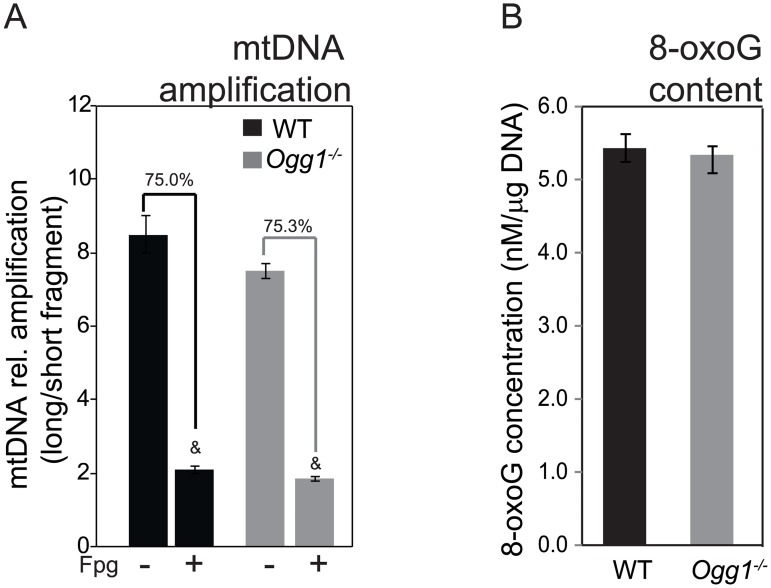
DNA damage estimations: mtDNA long amplicon PCR and total 8-oxoG content. A) Amplification of a long fragment of mtDNA was performed before and after treatment with FPG, a bacterial OGG1 functional analog. Data were normalized to amplification of a short mtDNA fragment. B) Total DNA was isolated by Miniprep and analyzed by ELISA for 8-oxoG content. Data are representative of 6 animals per genotype run in technical duplicates and expressed as mean ± SEM; ^&^, p<0.05 vs non-FPG treated.

mtDNA integrity was assessed by long-amplicon PCR, as previously described [50,51]. Amplification of a 10 kb mtDNA fragment before and after FPG treatment was normalized against amplification of a short mtDNA fragment. Differences in amplification before and after FPG treatment were interpreted to indicate sites of oxidatively modified bases. Using this assay, there were no measurable differences in mtDNA amplification between WT and *Ogg1*^*-/-*^ mice in skeletal muscle DNA (Fig 3A). These results are consistent with previous reports suggesting tissue-specific differences in accumulation and repair of oxidative DNA damage [9,52,53]. The lack of difference between WT and *Ogg1*^*-/-*^ mice is also supportive of the existence of backup repair mechanisms or alternative methods of dilution or elimination of mtDNA damage in skeletal muscle [9,54,55].

Interestingly, using this method, we observed a 75% reduction in amplification of mtDNA in both genotypes, upon FPG treatment. These data suggest that endogenous levels of oxidative lesions in post-mitotic tissues such as skeletal muscle may be rather high. Content of 8-oxoG in total DNA (nuclear and mtDNA) was measured using a commercially available ELISA. Total 8-oxoG content was not significantly different between WT and *Ogg1*^*-/-*^ mice (Fig 3B), indicating that under chow-fed conditions, there is no greater accumulation of 8-oxoG in skeletal muscle of *Ogg1*^*-/-*^ mice.

### Altered mitochondrial fission in *Ogg1*^*-/-*^ mice

Mitochondrial function is known to be affected not only by mitochondrial content, but also by mitochondrial morphology, which is maintained through constant fission and fusion processes [56–58]. We therefore measured two key markers of mitochondrial fission in muscle, *Drp1* and *Fis1*. Gene expression, as well as protein levels of these mediators of mitochondrial fission were both significantly increased in muscle of *Ogg1*^*-/-*^ mice, relative to WT counterparts (Fig 2C and 2D). These changes are consistent with previous reports regarding increased muscle mitochondrial fission being observed in obese and insulin-resistant muscle. However, it is notable that in *Ogg1*^*-/-*^ mice, these increases in markers of mitochondrial fission precede the development of obesity or insulin resistance, and may therefore be involved in the etiology of metabolic aberrations observed in these mice. Although the mechanisms leading to these changes in gene expression are not clear, some possible mechanisms are presented in the Discussion.

### Increased expression of lipid uptake genes in muscle of *Ogg1*^*-/-*^ mice

**Fig 4:**
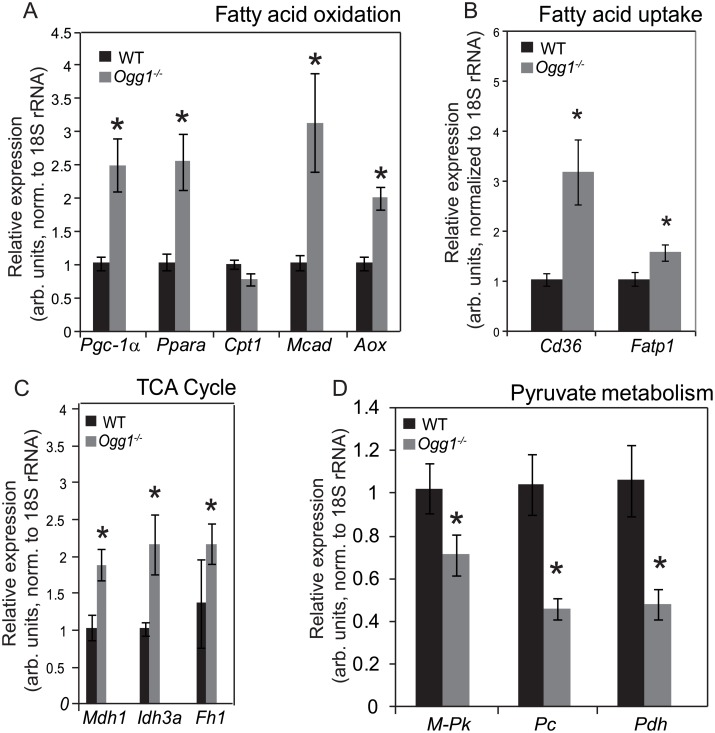
Expression of key metabolic genes. Genes regulating fatty acid oxidation (A) and uptake (B), as well as TCA cycle flux (C) and pyruvate metabolism (D) were measured by qPCR using gene-specific primers. Data are averages for 6 animals per genotypes and are expressed as mean ± SEM; *p<0.05 vs. WT. *Pgc1α*, Peroxisome proliferator-activated receptor gamma coactivator 1-alpha; *Pparα*, Peroxisome proliferator-activated receptor gamma coactivator 1-alpha; *Cpt1*, carnitine palmitoyl transferase-1; *Mcad*, medium-chain acyl-CoA dehydrogenase; *Aox*, acyl CoA oxidase; *Cd36*, cluster of differentiation 36; *Fatp1*, Fatty acid transport protein 1; *Mdh1*, malate dehydrogenase 1; *Idh3a*, isocitrate dehydrogenase 3 (NAD+) alpha; *Fh1*, fumarate hydratase 1; *M-Pk*, muscle pyruvate kinase; *Pc*, pyruvate carboxylase; *Pdh*, pyruvate dehydrogenase.

We have previously reported that genes regulating fatty acid oxidation (FAO) are significantly downregulated in livers of HFD-fed *Ogg1*^*-/-*^ mice, accompanied by increased hepatic lipid accumulation upon HFD-feeding [28]. To determine if the increased lipid deposition in skeletal muscle was accompanied by alterations in lipid oxidation, expression of key FAO genes was measured by qRT-PCR. Interestingly, *Ogg1*^*-/-*^ mice had increased expression of FAO genes (Fig 4A), relative to WT counterparts. Thus, the increased lipid deposition in muscle of chow-fed *Ogg1*^*-/-*^ mice is not likely to be a consequence of reduced fat oxidation. Levels of muscle lipids can be modulated not only by oxidation of fatty acids, but also by regulation of lipid uptake by fatty acid transporters [59–62]. Expression of the two key fatty acid transporters, fatty acid translocase (FAT/CD36) and fatty acid transport protein 1 (FATP1) was significantly upregulated in *Ogg1*^*-/-*^ mice, relative to WT counterparts (Fig 4B), commensurate with the increased muscle lipid deposition observed in these animals.

### Altered regulation of pyruvate metabolism and TCA cycle genes in *Ogg1*^*-/-*^ muscle

Since fatty acid uptake and oxidation genes were upregulated in *Ogg1*^*-/-*^ mice, we hypothesized that genes regulating TCA cycle flux may be differentially expressed in the muscle of these animals. Indeed, several key genes of the TCA cycle including malate dehydrogenase 1(*Mdh1*), isocitrate dehydrogenase 3a (*Idh3a*), and fumarate hydratase (*Fh1*) were significantly upregulated in *Ogg1*^*-/-*^ mice, relative to WT counterparts (Fig 4C). Conversely, expression of genes regulating synthesis and entry of the glycolytic endpoint, pyruvate, into the TCA cycle was significantly reduced in muscle of *Ogg1*^*-/-*^ mice. This included genes encoding muscle pyruvate kinase (*M-Pk*), which catalyzes the final step of glycolysis converting phosphoenolpyruvate to pyruvate; pyruvate carboxylase (*Pc*), which allows for entry of pyruvate into the TCA cycle via its conversion to oxaloacetate; and pyruvate dehydrogenase (*Pdh*), which catalyzes the conversion of pyruvate to acetyl-CoA as a substrate for the TCA cycle. These data collectively suggest that OGG1 deficiency results in altered skeletal muscle substrate metabolism, potentially favoring fat oxidation for meeting energy needs in muscle.

### Altered muscle function in *Ogg1*^*-/-*^ mice

**Fig 5:**
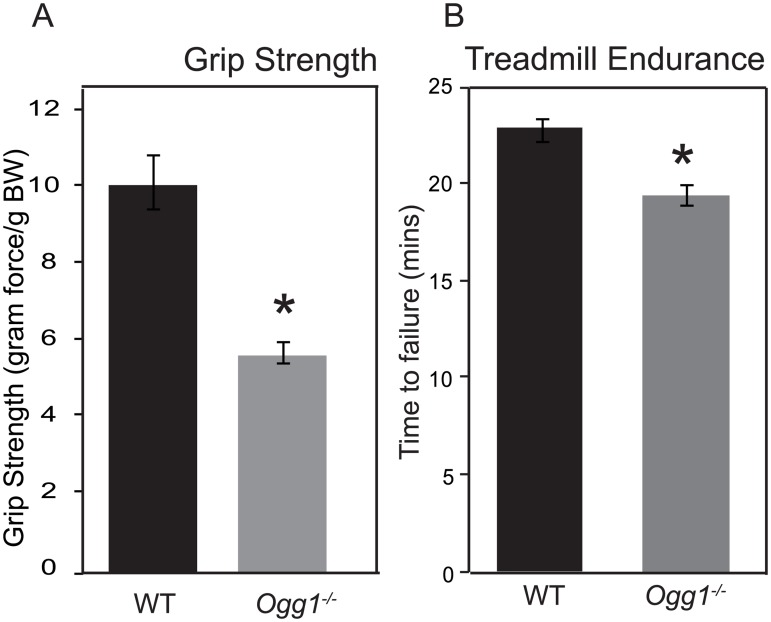
Alterations in muscle function. Muscle function was determined by measuring grip strength across all four limbs (A) and time to exhaustion on an elevated treadmill (B). Data are averages for 6 animals per genotypes and are expressed as mean ± SEM; *p<0.05 vs. WT.

To determine if these alterations in muscle carbohydrate and lipid metabolism translated to changes in muscle function, muscle grip strength and endurance were measured. Grip strength across all four limbs was significantly reduced in *Ogg1*^*-/-*^ mice, relative to WT counterparts (Fig 5A). In addition, when placed on a treadmill, *Ogg1*^*-/-*^ mice had significantly reduced times to exhaustion, when compared to WT counterparts, indicating decreased endurance in these animals (Fig 5B). Furthermore, we have previously reported that *Ogg1*^*-/-*^ mice have significantly reduced glucose tolerance [28]. Given that skeletal muscle is the primary site of glucose disposal, these data indicating reduced grip strength and endurance add to the evidence that OGG1 expression in skeletal muscle is essential for maintaining optimal muscle function.

## Discussion

We demonstrate here that OGG1 activity in skeletal muscle is an important determinant of muscle function and metabolism. Targeted deletion of this critical DNA repair enzyme results in marked alterations in skeletal muscle metabolism, favoring fat uptake and accumulation (Figs 1 and 5). We have shown previously that when placed on a HFD, *Ogg1*^*-/-*^ mice proceed to develop severe insulin resistance and other features of the metabolic syndrome, including fatty liver [28]. Interestingly, the changes in lipid metabolism and tissue function of skeletal muscle described herein precede the development of obesity or insulin resistance. They may therefore offer important insights into the etiology of metabolic disease in this model of oxidative stress-induced damage. It is notable that a recent study demonstrated that in a model of mitochondrial overexpression of OGG1, muscle myotubes were resistant to the lipotoxic effects of saturated fatty acids on insulin signaling [63], suggesting an important role for mitochondrial OGG1 in regulating metabolic health. While this may be the case in a model of OGG1 overexpression, the results of our mtDNA amplification assay (Fig 3A) suggest that under chow-fed basal conditions, there are no measurable differences between WT and *Ogg1*^*-/-*^ mice in the accumulation of FPG-sensitive lesions (primarily oxidized guanines) in muscle mtDNA. It was also notable that under chow-fed conditions, we observed no differences in total 8-oxoG content in skeletal muscle of *Ogg1*^*-/-*^ mice (Fig 3B). It is therefore plausible that the observed differences in skeletal muscle metabolism (Figs 4 and 5) stem from alternative functions of OGG1 such as its role in cell signaling and epigenetic regulation of gene expression [64–68].

### 8-oxoG as an epigenetic mark

Mechanistically, the mode of regulation of metabolic genes and genes of mitochondrial fission by OGG1 deficiency is not yet known. 8-oxoG in promoter regions of transcribed genes has been suggested to act as an epigenetic mark (rev. in [68]). For instance, binding of OGG1 to oxidized guanines in nuclear DNA has been shown to promote binding of transcription factors such as NFkB [66,67,69] and HIF1α [70]. This is notable since both *Drp1* and *Fis1* have been shown to be transcriptionally activated under conditions of HIF-1α activation [71]. In addition to the recruitment of transcription factors, OGG1 has been shown to participate in transcription initiation of estrogen-responsive genes, in which it is recruited through localized oxidative bursts stemming from active DNA demethylation at promoter sites [72]. Moreover, it has been recently reported that OGG1 recruits the DNA demethylase TET1 to sites of oxidative lesions, promoting DNA demethylation [73]. It is therefore possible that alterations in epigenetic marks, such as DNA methylation, may drive some of the gene expression changes observed in skeletal muscle of *Ogg1*^*-/-*^ mice.

### Linking OGG1 status to mitochondrial dynamics

*Ogg1*^*-/-*^ mice have significant alterations in markers of mitochondrial fission such that key genes and proteins regulating fission are significantly upregulated (Fig 2). Similarly, Torres-Gonzalez et al. recently reported that in a cultured cardiomyocyte model exposed to acute oxidative stress, adenoviral OGG1 overexpression resulted in reduced expression of DRP1 and FIS1 and decreased mitochondrial fragmentation [74], supporting the conclusion that OGG1 status is linked to regulation of mitochondrial dynamics in both *in vivo* and *in vitro* models. These data are especially interesting in light of a recent report suggesting that increased mitochondrial fission mechanistically contributes to the induction of insulin resistance in skeletal muscle [75]. This report by Jheng *et al*. demonstrated significant increases in muscle mitochondrial fission in models of obesity. However, the causal mechanisms leading to this increase were not determined.

In our model, the temporality of increased mitochondrial fission occurring prior to changes in body weight offers potentially important insights into the regulation of insulin sensitivity by mitochondrial fission. In the absence of efficient oxidative DNA repair, *Ogg1*^*-/-*^ mice have increased mitochondrial fission markers and subsequently go on to develop insulin resistance. This sequence of events is suggestive of OGG1 status being a determinant of mitochondrial dynamics. In this regard, it has been previously suggested that plasticity in mitochondrial dynamics may be a cellular tolerance mechanism for unrepaired damage. For instance, UV-induced helix-distorting lesions in mtDNA, which are not otherwise repaired through mitochondrial repair pathways, were shown to be tolerated via induction of both fission and fusion processes in a *C*. *elegans* model [76]. Although we did not observe any differences in static levels of oxidized lesions in mtDNA, as measured by the long-amplicon PCR method (Fig 3), it is possible that the increase in mitochondrial fission in *Ogg1*^*-/-*^ muscle (Fig 2C and 2D) serves to dilute damage as it is generated. Ongoing studies are designed to determine the consequences of pharmacological inhibition of mitochondrial fission in the context of OGG1 deficiency.

### Skeletal muscle lipid handling

In addition to these novel findings of altered mitochondrial fission, we also describe several changes in lipid content and composition in muscle of *Ogg1*^*-/-*^ mice. While it is known that high-fat feeding induces muscle lipid accumulation, it is interesting to note that OGG1 deficiency alone, even in the absence of a HFD, results in significant lipid accumulation in skeletal muscle (Fig 1). This increase in intramuscular lipids is accompanied by a significant increase in genes regulating fatty acid import into muscle cells (Fig 4). Muscle substrate utilization is primarily controlled by the availability of substrates. Under conditions of increased fatty acid availability, muscle fat uptake and oxidation is increased, while glucose oxidation is reduced. Conversely, under conditions of excess glucose, glucose oxidation is increased with lipid uptake and oxidation being reduced.

We previously reported that although lipid oxidation was reduced in livers of chow-fed *Ogg1*^*-/-*^ mice, there was no significant hepatic lipid accumulation under chow-fed conditions in these animals [28]. These data regarding muscle fatty acid handling therefore indicate that under chow-fed conditions, excess lipids may be channeled to skeletal muscle for utilization and storage. The increase in genes regulating both uptake and oxidation of fatty acids in muscle of chow-fed *Ogg1*^*-/-*^ mice may therefore be a consequence of increased lipid availability as a consequence of reduced hepatic lipid oxidation.

The finding of increased markers of fatty acid uptake in skeletal muscle of *Ogg1*^*-/-*^ mice is supportive of the idea that skeletal muscle may act as a sink for excess lipids in *Ogg1*^*-/-*^ animals, under chow-fed conditions. Given the known correlation between muscle lipid accumulation and insulin resistance, it is possible that this increase in lipids in skeletal muscle of *Ogg1*^*-/-*^ mice plays an important role both in modulation of mitochondrial dynamics (Fig 2), as well as in subsequent blunting of insulin sensitivity, as previously reported [28]. Furthermore, it is interesting to note that the reduction in arachidonate (20:4) in the PL fraction of *Ogg1*^*-/-*^ muscle is accompanied by an increase in arachidonate in the DAG fraction of these mice (Tables 5 and 3, respectively). The fatty acid composition of DAG has been shown to affect cellular outcomes such as activation of protein kinases, as well as signaling through the MAPK pathway via activation of RAS GTPases [77–79]. Given the observed differences in fatty acid composition of lipid moieties in *Ogg1*^*-/-*^ muscle, it may therefore be important to determine the role of these alterations in downstream signaling cascades in these mice.

The findings of increased lipid uptake in muscle of *Ogg1*^*-/-*^ mice also raise the question of lipid handling in muscle myotubes of OGG1 overexpressing mice. A previous report by Yuzefovych et al. indicated that muscle myotubes from mice overexpressing mitochondrial OGG1 were resistant to the toxic effects of palmitate exposure [63]. The effects of OGG1 overexpression on lipid uptake and metabolism were not determined in that study but would be interesting to measure in light of our results in *Ogg1*^*-/-*^ muscle (Fig 4B).

Based on the results of the current study, we propose that the absence of OGG1 alters both lipid metabolism, as well as mitochondrial health in skeletal muscle. While it is not known whether this occurs through OGG1's role in DNA repair or through its described roles in transcription regulation, it ultimately results in compromised muscle function and strength. These findings elucidate a novel pathway of metabolic regulation downstream of OGG1 and suggest an important role for this enzyme and the BER pathway in the maintenance of muscle function.

## Methods

### Animal studies

The generation of *Ogg1*^*-/-*^ mice backcrossed 21 times to C57BL/6J background has been previously described. [18,28] At OHSU, the *Ogg1*^*-/-*^ allele was maintained through additional backcrossing to C57BL/6J and subsequent matings between *Ogg1*^*+/-*^ mice. Age-matched male mice were exclusively used throughout this investigation. 12-week old mice were individually housed and given *ad libitum* access to rodent chow for 10 weeks prior to euthanasia. At the end of 10 weeks of feeding, mice were euthanized by CO_2_ overdose followed by cervical dislocation, and tissues were collected and snap frozen in liquid nitrogen for further analyses. For skeletal muscle experiments, mixed gastrocnemius tissue was used. For *in vivo* procedures, all efforts were made to minimize discomfort and suffering, in accordance with approved animal care protocols. The breeding and care of animals are in accordance with the protocols approved by the Animal Care and Use Committee of Oregon Health & Science University, Portland, Oregon.

### Tissue lipids

Total lipids were extracted from skeletal muscle according to the methods of Bligh and Dyer [80] and analyzed by TLC followed by gas chromatography, as described previously [81].

### Grip strength

Grip Strength of all 4 limbs was measured using a Chatillon E-DFE Series Digital Force Gauge. After a period of acclimatization, 7–8 mice of 18–20 weeks of age were analyzed 3 times sequentially, with a 7 minute rest between measurements. Data were recorded as gram/force exerted by the mouse and normalized for body weight.

### Treadmill running test

Mice were placed on the belt of a six-lane motorized treadmill (Exer 3/6 Treadmill; Columbus Instruments, Columbus, OH) supplied with shocker plates. The treadmill was run at an incline of 0° at 5 m/min for 5 min, after which the speed was increased 1 m/min every minute. The test was stopped when the mouse remained on the shocker plate for 20 s without attempting to reengage the treadmill, and the time to exhaustion was determined.

### mtDNA amplification assay

The long-amplicon quantitative PCR was performed as described [50,51] with few modifications. First, DNA was treated with FPG (New England Biolabs), which acts as an N-glycosylase and AP-lyase on 8-oxoG and oxidized purines such as 2,6-diamino-4-hydroxy-5-formamidopyrimidine (FapyG), and 4,6-diamino-5-formamidopyrimidine (FapyA) to generate PCR-blocking single strand breaks at sites of oxidative damage. Quantitative PCR was then used to determine the content of specific intact sequences. The difference in PCR amplification after FPG cleavage was compared to amplification in uncleaved DNA to estimate oxidative lesion content in mtDNA. Following FPG treatment, DNA was accurately quantified fluorometrically using PicoGreen (Quant-iT^™^ PicoGreen^®^ dsDNA Assay Kit, Invitrogen) and diluted in TE buffer (10 mM Tris-HCl, 1 mM EDTA, pH 8.0) to a final concentration of 3 μg/ml. Two targets of mtDNA were amplified: a long target to quantify levels of mtDNA lesions and a short target to control for changes in mtDNA copy number. The long target (≈ 10 kbp) spanned the *Nd1* and *Nd5* genes and the small target (127 bp) was part of *Nd1* gene. The primer nucleotide sequences were as follows: 5’-GCC AGC CTG ACC CAT AGC CAT AAT-3’ and 5’-GCC GGC TGC GTA TTC TAC GTT A-3’ for short target, 5’-GCC AGC CTG ACC CAT AGC CAT AAT-3’ and 5’-GAG AGA TTT TAT GGG TGT AAT GCG G-3’ for long target. The total volume for each PCR reaction was 20 μl, consisting of 15 ng of DNA template, 1x Platinum^™^ SuperFi^™^ PCR Master Mix (Invitrogen), 0.5 μM forward primer and 0.5 μM reverse primer. Samples were run in duplicates. The PCR parameters for the short mitochondrial target were 1 min at 95°C, then 16 cycles of 30 s at 95°C, 45 s at 72°C, followed by a final extension step of 5 min at 72°C. The parameters for the long mitochondrial target were 1 min at 95°C, then 15 cycles of 15 s at 95°C, 10 min at 70°C, followed by a final extension step of 10 min at 70°C. The PCR products were then quantified fluorometrically, and the results were expressed as mtDNA long amplicon amplification normalized for short amplicon amplification. Results before and after FPG treatment are shown.

### 8-oxoG ELISA

Total DNA was isolated from gastrocnemius of WT and *Ogg1*^*-/-*^ mice using the QIAamp DNA Mini Kit. Equal amounts of total DNA from each animal were pooled, and 10 μg of DNA was analyzed in duplicate using a commercially available 8-oxoG ELISA kit (Trevigen, Gaithersburg, MD) following manufacturer’s protocols.

### mtDNA quantitation and gene expression analysis

mtDNA content was measured by qPCR using primers directed against three different regions of mtDNA corresponding to regions encoding for ATP synthase F_o_ subunit 6 (*Atp6*), Cytochrome c oxidase subunit 2 (*CytoxII*), and NADH dehydrogenase 5 (*Nd5*). Data were normalized for the high-copy number nuclear gene, 18S rRNA. RNA for quantitative real-time PCR (qPCR) was isolated using Tri-reagent RT (MRC, Inc., Cincinnati, OH). 1 μg of RNA was reverse-transcribed using the Superscript III first-strand synthesis system (Invitrogen, Carlsbad, CA). qPCR was performed on a Bio-Rad iCycler qPCR instrument (Bio-Rad, Hercules, CA) using gene-specific primers. Gene expression was normalized to expression of 18S rRNA. Primer sequences are available upon request.

### Mitochondrial protein content

A total of 50 μg of mitochondrial proteins or whole cell lysates were resolved by SDS-PAGE followed by immunoblotting with antibodies against proteins of the mitochondrial oxidative phosphorylation (OXPHOS) complex and the voltage dependent anion channel (VDAC) (both from Mitosciences, Eugene, OR) or antibodies against mitochondrial fission 1 protein (FIS1) (Pierce, Waltham, MA), dynamin related protein 1 (DRP1) (Novus Biologicals, Littleton, CO), and glyceraldehyde 3-phosphate dehydrogenase (GAPDH) (Santa Cruz Biotechnology, Inc. Dallas, TX). Following incubation with IRDye secondary antibodies, proteins were visualized and quantified on a Li-COR imaging system (LI-COR Inc., Lincoln, NE).

### Statistical analyses

Data are expressed as mean ± SEM with comparisons carried out using a two-tailed student’s t-test for two-group comparisons using Graphpad Prism. p-values < 0.05 were considered significant.

## Supporting information

S1 Fig Full length blots of protein gels presented in Fig 2. (EPS)
